# Mental health impact of multiple sexually minoritized and gender expansive stressors among LGBTQ+ young adults: a latent class analysis

**DOI:** 10.1017/S2045796024000118

**Published:** 2024-04-11

**Authors:** C.-H. Shrader, J. P. Salerno, J.-Y. Lee, A. L. Johnson, A. B. Algarin

**Affiliations:** 1 Department of Epidemiology, Columbia University, New York City, NY, USA; 2 ICAP at Columbia University, New York City, NY, USA; 3 School of Social Work, Columbia University, New York City, NY, USA; 4 Department of Mental Health Law & Policy, University of South Florida, Tampa, FL, USA; 5 Department of Public Health Sciences, University of Miami School of Medicine, Miami, FL, USA; 6 Department of Nursing and Health Innovations, Arizona State University, Phoenix, AZ, USA

**Keywords:** minority stress, family rejection, internalized homophobia and transphobia, identity conealment, sexual and gender minority, psychological distress, college students

## Abstract

**Aims:**

In the United States, lesbian, gay, bisexual, transgender, queer, intersex, asexual and other sexually minoritized and gender expansive (LGBTQ+) young adults are at increased risk for experiencing mental health inequities, including anxiety, depression and psychological distress-related challenges associated with their sexual and gender identities. LGBTQ+ young adults may have unique experiences of sexual and gender minority-related vulnerability because of LGBTQ+-related minority stress and stressors, such as heterosexism, family rejection, identity concealment and internalized homophobia. Identifying and understanding specific LGBTQ+-related minority stress experiences and their complex roles in contributing to mental health burden among LGBTQ+ young adults could inform public health efforts to eliminate mental health inequities experienced by LGBTQ+ young adults. Therefore, this study sought to form empirically based risk profiles (i.e., latent classes) of LGBTQ+ young adults based on their experiences with familial heterosexist experiences, LGBTQ+-related family rejection, internalized LGBTQ+-phobia and LGBTQ+ identity concealment, and then identify associations of derived classes with psychological distress.

**Methods:**

We recruited and enrolled participants using nonprobability, cross-sectional online survey data collected between May and August 2020 (*N* = 482). We used a three-step latent class analysis (LCA) approach to identify unique classes of response patterns to LGBTQ+-related minority stressor subscale items (i.e., familial heterosexist experiences, LGBTQ+-related family rejection, internalized LGBTQ+-phobia and LGBTQ+ identity concealment), and multinomial logistic regression to characterize the associations between the derived classes and psychological distress.

**Results:**

Five distinct latent classes emerged from the LCA: (1) low minority stress, (2) LGBTQ+ identity concealment, (3) family rejection, (4) moderate minority stress and (5) high minority stress. Participants who were classified in the high and moderate minority stress classes were more likely to suffer from moderate and severe psychological distress compared to those classified in the low minority stress class. Additionally, relative to those in the low minority stress class, participants who were classified in the LGBTQ+ identity concealment group were more likely to suffer from severe psychological distress.

**Conclusion:**

Familial heterosexist experiences, LGBTQ+-related family rejection, internalized LGBTQ+-phobia and LGBTQ+ identity concealment are four constructs that have been extensively examined as predictors for mental health outcomes among LGBTQ+ persons, and our study is among the first to reveal nuanced gradients of these stressors. Additionally, we found that more severe endorsement of minority stress was associated with greater psychological distress. Given our study results and the previously established negative mental health impacts of minority stressors among LGBTQ+ young adults, findings from our study can inform research, practice, and policy reform and development that could prevent and reduce mental health inequities among LGBTQ+ young adults.

## Introduction

In the United States (U.S.), lesbian, gay, bisexual, transgender, queer, intersex, asexual and other sexually minoritized and gender expansive (LGBTQ+) young adults experience serious mental health burdens, including anxiety, depression and psychological distress (Ploderl and Tremblay, 2015; Fish et al., 2020; Valentine and Shiperd, 2018). LGBTQ+ young adults, including university students (who comprise 41% of young adults), may have unique experiences of sexual and gender minority-related oppression and vulnerability as a result of LGBTQ+-related minority stressors (Espinosa *et al.*, 2019; Gonzales *et al.*, 2020; Seelman *et al.*, 2017; Fish *et al.*, 2020). These can include family rejection, identity concealment and internalized homophobia (Newcomb and Mustanski, 2010; Pachankis *et al.*, 2020; Ryan *et al.*, 2009; Testa *et al.*, 2015). Thus, the mental health needs of LGBTQ+ young adults, including university students, warrants urgent attention and investigation.

LGBTQ+ university students experience elevated rates of minority stressors and psychological distress relative to non-LGBTQ+ students, with gender expansive university students experiencing unique psychological distress relative to their cisgender counterparts (Hunt *et al.*, 2021; Woodford *et al.*, 2018; Ploderl and Tremblay, 2015). Identifying and understanding LGBTQ+-related minority stressor-specific experiences and their complex roles in contributing to mental health burden among LGBTQ+ young adults and university students could inform public health efforts to eliminate psychological inequities, such as depression, anxiety and psychological distress, among these populations. Psychological distress is more widely defined as emotional suffering, which can include depressive symptomology (e.g., unhappiness) and anxiety (e.g., feeling tense) symptoms, and physical suffering (e.g., insomnia, headaches and lack of energy) (Belay *et al.*, 2021; Horwitz, 2002).

To better understand perceived oppression and stressors among LGBTQ+ young adults, the current study is guided by the Minority Stress Theory (Brooks, 1981; Meyer, 2003; Testa *et al.*, 2015). The Minority Stress Theory emphasizes the role of externalized (e.g., discrimination-related occurrences due to LGBTQ+ identity) and internalized (e.g., negative personal feelings related to LGBTQ+ identity) minority stressors and their impact on mental health among LGBTQ+ people. Previous studies have examined these constructs as independent variable-level predictors on various mental health outcomes, such as depression, anxiety and psychological distress (Chodzen *et al.*, 2019; Dyar *et al.*, 2020; Inderbinen *et al.*, 2021; McLean, 2021; Newcomb and Mustanski, 2010; Paceley *et al.*, 2020; Pachankis *et al.*, 2020; Puckett *et al.*, 2018). However, as these constructs do not exist in a vacuum, and instead, interact with each other, it is important to examine their intersections through use of non-linear modelling techniques, such as latent class analysis (LCA; Masyn, 2013; Weller *et al.*, 2020). Compared to the variable-centred general linear modeling, LCA, a person-centred model, could help elucidate the nuances of multiple LGBTQ+-related minority stress experiences, and how these experiences relate to psychological distress (Collins and Lanza, 2009; Richman and Lattanner, 2014). In other words, LCA can reveal hidden or unobservable groups of LGBTQ+ young adults based across their unique levels and combinations of multiple LGBTQ+-related minority stressors.

This study aimed to form empirically based risk profiles (i.e., latent classes) of LGBTQ+ young adults based on four minority stressors: familial heterosexist experiences, LGBTQ+-related family rejection, internalized LGBTQ+-phobia and LGBTQ+ identity concealment. Then, we aimed to identify which classes of LGBTQ+ young adults were at greater risk for experiencing psychological distress. We hypothesized that multiple latent classes would emerge from the data, and classes with higher minority stress would be associated with greater psychological distress. Findings from our study may inform research, practice, policy reform and development that could be used to prevent mental health challenges driven by minority stress among LGBTQ+ young adults.

## Methods

### Study design and sample

A nonprobability cross-sectional online survey was conducted between May 27 and August 14 2020. The aim of the parent study was to explore mental health and minority stress among LGBTQ+ university students. The University of Maryland provided institutional review board approval prior to study commencement. Additional study information can be found elsewhere (Salerno *et al.*, 2023).

An electronic recruitment flyer with a link to an online self-administered Qualtrics survey was distributed through popular social media platforms (i.e., Facebook, LinkedIn and Twitter) and email campaigning. Email campaigning included the university listservs of historically Black colleges and universities, Hispanic serving institutions and LGBTQ+ student centres across the U.S. Upon opening the survey online, participants completed a self-administered electronic informed consent process. Participant eligibility criteria included: (1) being a full-time student attending a U.S. tertiary institution, (2) identifying as LGBTQ+ and (3) being age 18 years or older. Participants were incentivized with the option to be entered into a raffle for a $50 Amazon electronic gift card.

### Measures

#### Socio-demographic characteristics

##### Gender identity

Participants indicated whether they identified as a *cisgender woman, a cisgender man, nonbinary, a transgender woman, transfeminine, a transgender man, transmasculine, genderqueer, two-spirit, gender fluid, agender* or *another gender*. Gender was recoded and categorized as cisgender man (referent), cisgender woman, non-binary, or genderqueer (included two-spirit, gender fluid, agender or other), transgender man or transmasculine, and transgender woman or transfeminine.

##### Age

Participants indicated their age in years.

##### Social isolation

Social isolation was measured using the three-item short loneliness scale (Hughes *et al.*, 2004). Item responses were coded on a 3-point Likert-type scale consisting of ‘hardly ever’ (1), ‘some of the time’ (2) and ‘often’ (3). We calculated the mean score of items to assess social isolation (*α* = 0.758).

##### Sexual orientation

Participants indicated their sexual orientation as *asexual, bisexual, gay, lesbian, same-gender loving, nonbinary, pansexual, queer, questioning, heterosexual/straight* or *another sexual identity.* Sexual orientation was recoded as bisexual/pansexual/non-binary (Flanders *et al.*, 2017), gay/lesbian/same-gender (Flanders *et al.*, 2017), queer, or another sexual identity (included heterosexual/straight, questioning and other) (Morandini *et al.*, 2017).

##### Race and ethnicity

Participants indicated their race (select all that apply) as *American Indian or Alaskan Native; Native Hawaiian or other Pacific Islander; Asian; Black or African American; White;* or *another race not listed*. Ethnicity was collected with the following yes/no question: ‘*Are you Hispanic or Latino?*’ Race and ethnicity were recoded and categorized as non-Hispanic White (referent); non-Hispanic Asian American Indian, non-Hispanic Alaskan Native, non-Hispanic Native Hawaiian or other Pacific Islander; non-Hispanic Black or African American; Latino or Hispanic, and multiracial or another race not listed.

#### LGBTQ+-related minority stress latent class indicator variables

The LGBTQ+-related minority stress survey items can be found in Appendix 1. For the LCA, items were assessed individually.

##### Familial heterosexist experiences

An adapted version of seven items from the Daily Heterosexist Experiences Questionnaire (DHEQ) ‘Family of Origin’ subscale (Balsam *et al.*, 2013) was used to assess past-year experiences of heterosexism perpetrated by family members. To capture presence of past year familial heterosexist experiences, students were asked to indicate if they experienced these stressors in the past year (yes = 1; no = 0). For descriptive analysis, a composite score was calculated by summing responses across the seven items (*α* = 0.729).

##### LGBTQ+-related family rejection

An adapted version of 10 items from the ‘family rejection’ subscale of the Sexual Minority Adolescent Sexual Minority Stress Inventory (Schrager *et al.*, 2018) was used to measure past year LGBTQ+-related family rejection. To capture presence of past year LGBTQ+-related family rejection, students were asked to indicate if they experienced these stressors in the past year (yes = 1; no = 0). For descriptive analysis, a composite score was calculated by summing responses across the 10 items (*α* = 0.821).

##### Internalized LGBTQ+-phobia

An adapted version of seven items from the LGBT Minority Stress Measure (LMSM; Outland, 2016) was used to measure past year internalized LGBTQ+-phobia. To capture presence of past year internalized LGBTQ+-phobia, students were asked to indicate if they experienced these stressors in the past year (yes = 1; no = 0). A composite score was calculated by summing responses across the seven items (*α* = 0.801).

##### LGBTQ+ identity concealment

LGBTQ+ identity concealment within the past year was measured using an adapted version of three items from the LMSM (Outland, 2016) and four items from the DHEQ (Balsam *et al.*, 2013). To capture presence of past year LGBTQ+ identity concealment, students were asked to indicate whether they experienced these stressors in the past year (yes = 1; no = 0). A composite score was calculated by summing responses across the seven items (*α* = 0.768).

##### Psychological distress

The previously validated 10-item Kessler-10 (K10) was used to measure current nonspecific psychological distress (Kessler *et al.*, 2002). This 10-item scale provided measures of depression and anxiety within the past 30 days. Item responses were coded on a 5-point Likert-type scale from ‘none of the time’ (1) to ‘all of the time’ (5). There was strong internal consistency for psychological distress in the current sample (*α* = 0.801). Participants were classified as having ‘healthy’ (referent), ‘mild’, ‘moderate’ or ‘severe’ psychological distress (Andrews and Slade, 2001; Slade *et al.*, 2011).

##### Analytic framework

Using a person-centred approach, we used LCA to form empirically based risk profiles of LGBTQ+ young adults based on their response patterns to LGBTQ+ minority stress subscale items (i.e., familial heterosexist experiences, LGBTQ+-related family rejection, internalized LGBTQ+-phobia and LGBTQ+ identity concealment) (Fergusson *et al.*, 2005). A total of 31 binary minority stress variable items were assessed in the LCA. Using the *poLCA* package on the R environment (Lewis and Linzer, 2011; R Core Team, 2013), we conducted LCA with two to six classes. Due to sample size limitations, we opted not to include covariates in the LCA and instead utilize covariates in the multinomial logistic regression. We chose to use 30 repetitions to estimate the LCA model and used random matrices of class-conditional response probabilities as the starting values. We set the LCA to run a maximum of 3,000 iterations. We used the following fit statistics to assess which model solution best fit our data: class sizes, intra-class correlations, average posterior probabilities, consistent Akaike’s information criterion (cAIC), Bayes information criterion (BIC), Akaike’s Bayes information criterion (aBIC), Lo–Mendell–Rubin likelihood ratio test (LMR), bootstrap likelihood ratio test (LRT) and entropy. Participants with missing data across the 31 minority stress items were removed from analyses (1.8% missing).

To test class differences between LCA class assignment and psychological distress, chi-square tests of association were used. To test for class differences between LCA class assignment and minority stress items, we used analysis of variance. To test for multivariable (adjusted for socio-demographic characteristics) associations between latent class assignment and psychological distress, we used multinomial logistic regression. Alpha was set to 0.05, and all bivariate and multivariable statistical models were conducted using the *nnet* package using R Statistical Software (R Core Team, 2013; Ripley *et al.*, 2016).

## Results

### Socio-demographic findings

A descriptive summary of total sample (*N* = 482) and class-specific socio-demographic characteristics are described in Table 1. Participants reported a mean age of 22 years, and most participants identified as cisgender women (54%), non-Hispanic (85%), non-Hispanic White (70%) and single (51%).

**Table 1.** Sample socio-demographic and background characteristics stratified by latent class, *N* = 482

	Low minority stress (*n* = 119)	LGBTQ identity concealment (*n* = 133)	Family rejection (*n* = 109)	Moderate minority stress (*n* = 61)	High minority stress (*n* = 60)	Overall	Test statistic
Mean age (SD)**	22.9 (4.21)	21.3 (3.34)	21.7 (4.24)	22.9 (4.79)	21.2 (2.99)	22.1 (4.06)	*F* = 4.5
**Race and ethnicity^a^**							*χ*^2^ = 22.0
White	78 (65.5%)	76 (57.1%)	35 (58.3%)	66 (60.6%)	37 (60.7%)	292 (60.6%)	
Asian/AI/NHOPI	13 (10.9%)	21 (15.8%)	7 (11.7%)	7 (6.4%)	7 (11.5%)	55 (11.4%)	
Black/African American	5 (4.2%)	8 (6.0%)	6 (10.0%)	12 (11.0%)	8 (13.1%)	39 (8.1%)	
Hispanic/Latinx	17 (14.3%)	12 (9.0%)	10 (16.7%)	15 (13.8%)	7 (11.5%)	61 (12.7%)	
Multiracial or another race^b^	6 (5.0%)	16 (12.0%)	2 (3.3%)	9 (8.3%)	2 (3.3%)	35 (7.3%)	
**Gender identity**							*χ*^2^ = 15.0
Cisgender man	17 (14.3%)	11 (18.3%)	6 (9.8%)	19 (17.4%)	24 (18.0%)	77 (16.0%)	
Cisgender woman	68 (57.1%)	30 (50.0%)	37 (60.7%)	53 (48.6%)	73 (54.9%)	261 (54.1%)	
Non-binary or genderqueer	24 (20.2%)	13 (21.7%)	13 (21.3%)	25 (22.9%)	28 (21.1%)	103 (21.4%)	
Trans man/masculine	7 (5.9%)	6 (10.0%)	5 (8.2%)	10 (9.2%)	3 (2.3%)	31 (6.4%)	
Trans woman/feminine	3 (2.5%)	0 (0%)	0 (0%)	2 (1.8%)	5 (3.8%)	10 (2.1%)	
**Sexual identity**							*χ*^2^ = 26.2
Bisexual, pansexual or non-binary	52 (43.7%)	45 (41.3%)	24 (40.0%)	30 (49.2%)	57 (42.9%)	208 (43.2%)	
Asexual	8 (6.7%)	5 (4.6%)	4 (6.7%)	4 (6.6%)	14 (10.5%)	35 (7.3%)	
Gay, lesbian or same-gender loving	43 (36.1%)	36 (33.0%)	26 (43.3%)	14 (23.0%)	42 (31.6%)	161 (33.4%)	
Queer	15 (12.6%)	23 (21.1%)	3 (5.0%)	13 (21.3%)	18 (13.5%)	72 (14.9%)	
Another sexual identity^c^	1 (0.8%)	3 (5.0%)	0 (0%)	0 (0%)	2 (1.5%)	6 (1.2%)	
Mean social isolation score (SD)***	1 (0.8%)	0 (0%)	3 (5.0%)	0 (0%)	2 (1.5%)	6 (1.2%)	*F* = 9.2

a All race/ethnicity categories other than Hispanic/Latino refer to non-Hispanic/Latinx participants.

b Another race included Arab.

c Another sexual identity includes questioning or heterosexual/straight.

** indicates significance at the *p* < 0.01 level; ***indicates significance at the *p* < 0.001 level.

### Latent class analysis

**Figure 1.**
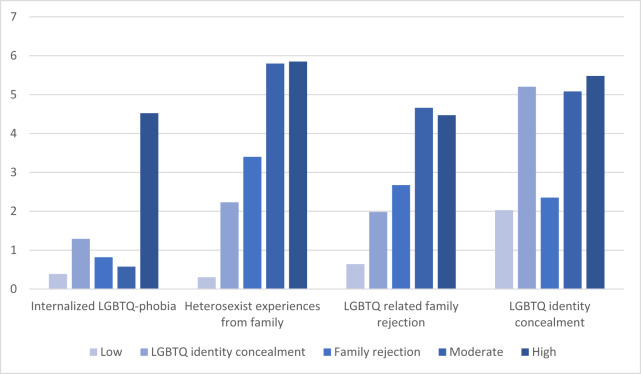
Composite minority stress indicator variables stratified by latent class group.

Latent class analysis model fit indices are reported in Table 2. All five classes in the five-class model demonstrated adequate sample sizes that met the suggested 10% of the total sample threshold (*n* = 119, *n* = 133, *n* = 109, *n* = 61, *n* = 60) (Sinha *et al.*, 2021). The five-class model produced the lowest AIC, BIC and aBIC compared to all models and detected significant LMR compared to the four-class model, suggesting that this is the strongest model in-terms of cAIC, BIC, and aBIC (four-class model not further considered). The entropy of the five-class model was above the 0.80 recommended cut-off (Nylund-Gibson and Choi, 2018; Weller *et al.*, 2020), suggesting composition of classes with strong separation (Nylund-Gibson and Choi, 2018). We then examined the five-class model for interpretability and discovered a meaningful pattern (Fig. 1). Therefore, the five-class model was determined to be conceptually interpretable, with strong model fit, and was selected as the final model for further analysis.

**Table 2.** Results of the latent class analysis enumeration and model fit indices for two to six classes

Classes	cAIC	BIC	aBIC	Max log-likelihood	Residual degrees of freedom	Likelihood-ratio	BLRT *p*-value	Entropy	LMR-LRT	LMR-LRT *p*-value
2	14,865.90	14,802.90	14,602.94	−7206.71	421	8591.39	<0.0001	0.86	–	–
3	14,865.90	14,802.90	14,602.94	−7206.71	421	8591.39	<0.0001	0.89	556.802	<0.001
4	14,508.90	14,413.90	14,112.37	−6913.30	389	8004.57	<0.0001	0.89	426.871	<0.001
5	14,288.84	14,161.84	13,758.75	−6688.36	357	7554.68	<0.0001	0.89	199.573	<0.001
6	14,308.33	14,149.33	13,644.68	−6583.19	325	7344.34	<0.0001	0.88	186.356	<0.001

cAIC = consistent Akaike’s information criterion, BIC = Bayes information criterion, aBIC = Akaike’s Bayes information criterion, BSLRT = bootstrap likelihood ratio test; LMR-LRT = Lo–Mendell–Rubin likelihood ratio test.

Because the five-class model is the most parsimonious model, participants were categorized into five distinct minority stress classes: low minority stress (*n* = 119), LGBTQ+ identity concealment only (*n* = 133), family rejection only (*n* = 109), moderate minority stress (*n* = 61) and high minority stress (*n* = 60). A descriptive summary of minority stressor means stratified by class membership is reported in Table 3 and visualized in Fig. 1 (using composite scores). There were statistically significant differences in class membership and age (*p* < 0.001) and mean social isolation score (*p* < 0.001) based on minority stress latent class assignment.

**Table 3.** Latent class analysis minority stressor indicator variables as composite scores, stratified by latent class, N = 482

	Low minority stress	LGBTQ identity concealment	Family rejection	Moderate minority stress	High minority stress	Overall	Test statistic
Internalized LGBTQ-phobia***							*F*-value = 159.3
Mean (SD)	0.387 (0.760)	1.29 (1.46)	0.817 (1.08)	0.574 (0.763)	4.52 (1.11)	1.27 (1.68)	
Median [Min, Max]	0 [0, 3.00]	1.00 [0, 6.00]	0 [0, 4.00]	0 [0, 2.00]	5.00 [2.00, 6.00]	1.00 [0, 6.00]	
Heterosexist experiences from family***							*F*-value = 282.4
Mean (SD)	0.303 (0.590)	2.23 (1.40)	3.40 (1.59)	5.80 (1.40)	5.85 (1.44)	2.92 (2.39)	
Median [Min, Max]	0 [0, 3.00]	2.00 [0, 6.00]	3.00 [0, 8.00]	6.00 [2.00, 8.00]	6.00 [4.00, 8.00]	3.00 [0, 8.00]	
LGBTQ related family rejection***							*F*-value = 142.1
Mean (SD)	0.639 (0.810)	1.98 (0.945)	2.67 (1.43)	4.66 (1.55)	4.47 (2.05)	2.45 (1.92)	
Median [Min, Max]	0 [0, 3.00]	2.00 [0, 5.00]	2.00 [0, 7.00]	5.00 [2.00, 8.00]	4.00 [1.00, 9.00]	2.00 [0, 9.00]	
LGBTQ identity concealment***							*F*-value = 172.4
Mean (SD)	2.03 (1.62)	5.20 (1.04)	2.35 (1.21)	5.08 (1.14)	5.48 (1.21)	3.79 (1.99)	
Median [Min, Max]	2.00 [0, 6.00]	5.00 [3.00, 7.00]	2.00 [0, 5.00]	5.00 [3.00, 7.00]	5.00 [3.00, 7.00]	4.00 [0, 7.00]	

*** indicates significance at the *p* < 0.001 level.

The low minority stress class was characterized by participants having low conditional probability (i.e., Pr ∼ 0.1) of responding ‘yes’ to most items across all minority stress subscales.

The LGBTQ+ identify concealment class was characterized by participants having moderately high conditional probability (i.e., 0.5 < Pr < 1) of responding ‘yes’ to the LGBTQ+ identity concealment items and moderately low conditional probability (i.e., Pr < 0.3) of responding ‘yes’ to all other items.

The family rejection only class was characterized by participants having a moderately low conditional probability (i.e., 0.1 < Pr < 0.3) of responding ‘yes’ to most items, with the exception of the LGBTQ+-related family rejection subscales in which participants were characterized by moderate conditional probability (i.e., 0.3 > Pr > 0.7) of responding ‘yes’ to the majority of items.

The moderate minority stress class was characterized by participants having a high (i.e., 0.5 > Pr > 1) conditional probability of responding ‘yes’ to the majority of items in the LGBTQ+-related family rejection and LGBTQ+ identity concealment subscales, a moderate probability of responding ‘yes’ to the majority familial heterosexist experiences items (i.e., Pr ∼ 0.4) and low conditional probability of responding ‘yes’ to the majority of items in the internalized LGBTQ+-phobia subscale (i.e., Pr ∼ 0.1).

The high minority stress class was characterized by participants having a high (i.e., 0.5 > Pr > 1) conditional probability of responding ‘yes’ to most items across all subscales.

### Bivariate latent class group differences in psychological distress

Frequencies of participants’ psychological distress levels stratified by class membership and bivariate associations between psychological distress and class membership are found in Table 4. Most participants in the high minority stress class (70%) demonstrated severe psychological distress. Approximately half of participants in the LGBTQ+ identity concealment class (47%) and moderate minority stress class (51%) demonstrated severe psychological distress. Approximately, 38% of participants in the family rejection only class demonstrated severe psychological distress. Of participants in the low minority stress class, 25% indicated demonstrated severe psychological distress. We identified a statistically significant association between latent class membership and psychological distress (*χ*^2^ = 45.78, *p* < 0.001).

**Table 4.** Psychological distress of LGBTQ+ university students, stratified by latent class, N = 482

	Low	LGBTQ identity concealment	Family rejection	Moderate	High	Overall (*N* = 482)	Test statistic
Psychological distress***							*χ*^2^ = 46.0
Healthy	30 (25.2%)	23 (17.3%)	20 (18.3%)	5 (8.2%)	3 (5.0%)	81 (16.8%)	
Mild psychological distress	33 (27.7%)	19 (14.3%)	22 (20.2%)	11 (18.0%)	4 (6.7%)	89 (18.5%)	
Moderate psychological distress	26 (21.8%)	29 (21.8%)	26 (23.9%)	14 (23.0%)	11 (18.3%)	106 (22.0%)	
Severe psychological distress	30 (25.2%)	62 (46.6%)	41 (37.6%)	31 (50.8%)	42 (70.0%)	206 (42.7%)	

*** indicates significance at the *p* < 0.001 level.

### Multivariable associations between latent class membership and psychological distress

Table 5 describes the results of the multinomial logistic regression analysis testing the multivariable associations between latent class membership and psychological distress (adjusting for socio-demographic characteristics).

**Table 5.** Multivariable multinomial logistic regression testing the associations between latent class group and psychological distress

**Mild psychological distress**				**Moderate psychological distress**				**Severe psychological distress**			
	***OR***	***CI***	***p***		***OR***	***CI***	***p***		***OR***	***CI***	***p***
**(Intercept)**	0.11	0.01–1.44	0.092	**(Intercept)**	0.71	0.05–10.91	0.803	**(Intercept)**	0.00	0.00–0.05	**<0.001**
**Minority stress LCA profile** **(ref = low)**				**Minority stress LCA profile** **(ref = low)**				**Minority stress LCA profile** **(ref = low)**			
LGBTQ identity concealment	0.78	0.34–1.82	0.567	LGBTQ identity concealment	1.55	0.67–3.58	0.304	LGBTQ identity concealment	2.78	1.23–6.32	**0.014**
Moderate minority stress	1.06	0.47–2.40	0.883	Moderate minority stress	1.52	0.66–3.49	0.323	Moderate minority stress	1.99	0.86–4.57	0.106
Family rejection	2.40	0.70–8.19	0.163	Family rejection	3.47	1.01–11.87	**0.048**	Family rejection	8.29	2.50–27.50	**0.001**
High minority stress	1.53	0.29–8.03	0.613	High minority stress	5.47	1.22–24.48	**0.026**	High minority stress	15.25	3.65–63.71	**<0.001**
**Age**	1.03	0.95–1.12	0.492	**Age**	0.94	0.86–1.04	0.238	**Age**	1.08	1.00–1.17	**0.047**
**Race (White)^a^**				**Race (White)^a^**				**Race (White)^a^**			
Asian/AI/NHOPI	0.60	0.25–1.46	0.258	Asian/AI/NHOPI	0.22	0.08–0.60	**0.003**	Asian/AI/NHOPI	0.42	0.17–1.02	0.054
Black or African American	0.39	0.12–1.31	0.128	Black or African American	0.44	0.15–1.27	0.127	Black or African American	0.63	0.22–1.78	0.378
Hispanic or Latino	0.76	0.30–1.95	0.563	Hispanic or Latino	0.40	0.14–1.10	0.075	Hispanic or Latino	1.14	0.47–2.77	0.771
Multiracial/other race	3.51	0.38–32.09	0.266	Multiracial/ other race	2.37	0.26–21.68	0.444	Multiracial/other race	8.71	1.07–70.62	**0.043**
**Gender (ref = cisgender man)**				**Gender (ref = cisgender man)**				**Gender (ref = cisgender man)**			
Cisgender woman	2.75	1.26–5.97	**0.011**	Cisgender woman	3.14	1.37–7.17	**0.007**	Cisgender woman	5.66	2.51–12.74	**<0.001**
Nonbinary or genderqueer	1.72	0.57–5.19	0.336	Nonbinary or genderqueer	6.36	2.26–17.92	**<0.001**	Nonbinary or genderqueer	9.43	3.41–26.08	**<0.001**
Transgender man/transmasculine	1.31	0.25–6.88	0.747	Transgender man/transmasculine	4.59	1.08–19.46	**0.039**	Transgender man/transmasculine	9.49	2.28–39.58	**0.002**
Transgender woman/transfeminine	2.13	0.26–17.81	0.484	Transgender woman/transfeminine	1.16	0.09–15.47	0.909	Transgender woman/transfeminine	8.91	1.14–69.50	**0.037**
**Social isolation score**	1.78	0.99–3.20	0.053	**Social isolation score**	1.47	0.81–2.65	0.202	**Social isolation score**	5.98	3.32–10.76	**<0.001**
Observations	482										
*R*^2^ Nagelkerke	0.150/0.148										

a All race/ethnicity categories other than Hispanic/Latino refer to non-Hispanic/Latinx participants.

The bold values indicate the variable name/group.

#### Mild psychological distress

Participants who identified as cisgender women were more likely to experience mild psychological distress compared to cisgender men (OR = 2.75; 95% CI: 1.26–5.97; *p* = 0.011).

#### Moderate psychological distress

Membership in the high minority stress class (relative to the low minority stress class; OR = 5.47; 95% CI: 1.22–24.48; *p* = 0.026) and in the moderate minority stress class (relative to the low minority stress class; OR = 3.47; 95% CI: 1.01–11.87; *p* = 0.048) was associated with greater likelihood of experiencing moderate psychological distress. Further, participants who identified as non-Hispanic Asian, American Indian, or Native Hawaiian and Pacific Islander (relative to non-Hispanic White; OR = 0.22; 95% CI: 0.08–0.60; *p* = 0.003) were less likely to experience moderate psychological distress. Participants who identified as cisgender women (OR = 3.14; 95% CI: 1.37–7.17; *p* = 0.007), non-binary or genderqueer (OR = 6.36; 95% CI: 2.26–17.92; *p* < 0.001) or transgender men/transmasculine (OR = 4.59; 95% CI: 1.08–19.46; *p* = 0.039), relative to cisgender men, were more likely to experience moderate psychological distress.

#### Severe psychological distress

Membership in the high minority stress class (relative to the low minority stress class; OR = 15.25; 95% CI: 3.65–63.71; *p* < 0.001), moderate minority stress class (OR = 8.29; 95% CI: 2.50–27.50; *p* = 0.001) and LGBTQ+ identity concealment class (OR = 2.78; 95% CI: 1.23–6.32; *p* = 0.014) was associated with greater likelihood of experiencing severe psychological distress. Greater age was associated with increased likelihood of experiencing severe psychological distress (OR = 1.08; 95% CI: 1.00–1.17; *p* = 0.047). Participants who identified as multiracial or another race (relative to non-Hispanic White; OR = 8.71; 95% CI: 1.07–70.62; *p* = 0.043), a cisgender woman (relative to cisgender man; OR = 5.66; 95% CI: 2.51–12.74; *p* < 0.001), non-binary or genderqueer (relative to cisgender man; OR = 9.43; 95% CI: 3.41–26.08; *p* < 0.001), a transgender man/transmasculine (relative to cisgender man; OR = 9.49; 95% CI: 2.28–39.58; *p* = 0.002) and a transgender woman/transfeminine (relative to cisgender man; OR = 8.91; 95% CI: 1.14–69.50; *p* = 0.037) were more likely to experience severe psychological distress. Lastly, greater social isolation (OR = 5.98; 95% CI: 3.32–10.76; *p* < 0.001) was associated with increased likelihood of severe psychological distress.

## Discussion

This study identified unique groups of LGBTQ+ young adults based on their differential experiences of LGBTQ+-related minority stress across five classes: low, LGBTQ+ identity concealment, family rejection only, moderate and high minority stress. Our hypothesis was partially correct; latent class membership was associated with severity of psychological distress, such that those in the moderate minority stress and high minority stress groups were consistently at increased risk for moderate and severe psychological distress compared to the low minority stress group, and the LGBTQ+ identity concealment group was at increased risk for severe psychological distress compared to the low minority stress group. However, our hypothesis was also partially incorrect: Asian, American Indian, or Native Hawaiian and Pacific Islander (compared to non-Hispanic White) LGBTQ+ university students were less likely to suffer from moderate psychological distress, and multiracial or another race identifying LGBTQ+ university students were more likely to suffer from severe psychological distress. Our study reveals the salient impact of multiple LGBTQ+-related minority stress on psychological distress among LGBTQ+ young adults. Our study is among the first to demonstrate that nuanced gradients of minority stress were associated with greater likelihood of psychological distress among LGBTQ+ young adults.

Findings around the high and moderate minority stress groups are consistent with existing literature documenting the negative effects of minority stress on LGBTQ+ young adults’ mental health (Price-Feeney *et al.*, 2020; Newcomb and Mustanski, 2010; Pachankis *et al.*, 2020; Ryan *et al.*, 2009; Testa *et al.*, 2015) and suggest an additive or perhaps compacting or intersecting relationship, in which more minority stress correlates with greater magnitude of psychological distress, supporting our hypothesis. Yet, findings on the LGBTQ+ identity concealment group reveal that this stressor may have a particularly strong impact on mental health among young adults (Pachankis *et al.*, 2020), even when other stressors such as family rejection, familial heterosexist experiences and internalized LGBTQ+-phobia are at lower levels. Lastly, the fact that the LGBTQ+-related family rejection only group did not demonstrate significance for any elevated level of psychological distress counters previous evidence documenting the salient impact of family rejection on the mental health of LGBTQ+ youth (Gattamorta *et al.*, 2022; Klein and Golub, 2016; Mitrani *et al.*, 2017; Ryan *et al.*, 2009). This unexpected finding could relate to analytical, measurement or sample differences compared to past studies and calls for more nuanced conceptualization of LGBTQ+-related family rejection scales and more application of complex and non-linear models of minority stress.

We found that Asian, American Indian, and Native Hawaiian or Pacific Islander (compared to non-Hispanic White) identifying LGBTQ+ university students were less likely to experience moderate psychological distress. Our findings suggest the possibility that these populations are resilient in resolving psychological distress relative to their non-Hispanic White counterparts or perhaps are less likely to recognize their psychological distress due to the stigma surrounding mental health in these communities (Misra *et al.*, 2021; Ng, 1997). We also discovered that LGBTQ+ university students who identified as multiracial or other another race were more likely to experience severe psychological distress compared to non-Hispanic White LGBTQ+ university students. This may be related to their unique intersectional experiences of discrimination and racism (Bowleg *et al.*, 2003; Salerno *et al.*, 2023) along with multiple LGBTQ+-related minority stress experiences. It is imperative for researchers to investigate the intersectionality of race, ethnicity and sexually minoritized and gender expansive identities to address mental health concerns among these populations. Future research is needed to understand how racial and ethnic identities create risk or resilience and how to leverage these identities to prevent and address LGBTQ+-related minority stressors and negative mental health outcomes among LGBTQ+ young people.

As this LCA has indicated, psychological health inequities are driven by LGBTQ+-related minority stressors. As such, it is important for treatment to address co-occurring, additive and compounding LGBTQ+-related minority stress. Recent research identified 44 individual-, interpersonal-, structural- and multi-level interventions developed to reduce sexual minority stressors and/or bolster coping resources and strategies (Chaudoir *et al.*, 2017). For instance, Puckett and Levitt’s general guidelines include aiming to understand LGBTQ+ clients’ minority stress in the context of the oppressive U.S. system, not overattributing mental health symptoms to internalized stigma, and helping LGBTQ+ clients recognize when minority stress affects their mental health (Puckett and Levitt, 2015). Future minority stress and psychosocial distress reduction programs could examine how to tailor psychosocial and multicomponent strategies based on the intersections of multiple LGBTQ+-related minority stressors, such as those identified among participants in the moderate and high minority stress classes. Intersectionality framing of mental health and minority stress interventions for LGBTQ+ young adults could be beneficial in this regard (Huang *et al.*, 2020). Interdisciplinary, public health and mental health scientists and practitioners are needed urgently to improve understanding of how to adapt existing culturally relevant resources to prevent mental illness driven by multiple LGBTQ+-related minority stress among LGBTQ+ young adults.

This study had several limitations. This study used a non-probability sampling strategy, which limits our ability to generalize findings to broader populations of LGBTQ+ young adults. As a cross-sectional study, responses were subject to recall bias, and we were unable to test causality and temporality among constructs; such limitations are important to consider in the context of intervention development. Due to correlations between variables, sample size limitations, and the existing complexity of this analysis, we were unable to utilize an intersectional perspective to investigate the significance of multiply marginalized identities within LGBTQ+ young adults, which includes sexual identity. This should be addressed in future studies with additional resources and greater sample sizes. Lastly, our survey collected data surrounding LGBTQ+ young adults’ experiences of minority stress during the COVID-19 pandemic, which may be a factor that influences our study findings, as participants may have been under greater stress during the pandemic, as seen in other studies (Wang *et al.*, 2020). However, our results add to emerging literature, which indicates that LGBTQ+ people are experiencing mental health disparities and treatment access inequities since the start of the COVID-19 pandemic (Algarin *et al.*, 2022; Kamal *et al.*, 2021; Salerno and Boekeloo, 2022). Despite limitations, this study provides important public health implications to consider for the mental health of LGBTQ+ young adults.

Our study findings have important public health implications for LGBTQ+ young adults. First, mental health services could address intersecting and multidimensional LGBTQ+-related minority stress in their practices and policies to mitigate poor mental health among LGBTQ+ young adults (Huang *et al.*, 2020). Recommendations for mental health services include increasing access to LGBTQ+-affirming mental health care (Austin *et al.*, 2018; Burton *et al.*, 2019; Chaudoir *et al.*, 2017; Cohen *et al.*, 2018; Hughto *et al.*, 2019; Pachankis, 2015; Ryan, 2009) and dissemination of resources useful for the prevention of LGBTQ+-related minority stress and psychological distress (Cohen *et al.*, 2018; Diamond and Shpigel, 2014; Ryan, 2009; SAMHSA, 2014). Despite significant advancement in the development of mental health and minority stress reduction programs for LGBTQ+ youth (IOM, 2011; Romanelli and Hudson, 2017), there is a severe dearth of competent and affirming mental health services and providers equipped with the tools, resources and skills to meet the needs of LGBTQ+ young adults in the U.S. (Williams and Fish, 2020). This is highly concerning given that LGBTQ+ young adults are already less likely to use mental health services compared to their heterosexual and cisgender counterparts (Bourdon *et al.*, 2020; Dunbar *et al.*, 2017; Filice and Meyer, 2018; Progovac *et al.*, 2018). Indeed, significant barriers in access to treatment among LGBTQ+ young adults have been identified (Chaudoir *et al.*, 2017; IOM, 2011; Romanelli and Hudson, 2017).

To advance the prevention of severe mental health burdens driven by LGBTQ+-related minority stress, it is imperative to increase the wide implementation and dissemination of LGBTQ+ affirmative practice and to prepare a mental health workforce that is able to address the unique identity-related concerns of LGBTQ+ young adults. Familial heterosexist experiences, LGBTQ+-related family rejection, LGBTQ+ identity concealment and internalized LGBTQ+-phobia are four constructs that have been extensively examined under the minority stress theory to serve as predictors for mental health outcomes among LGBTQ+ persons. Given our study results and the previously established negative mental health impacts of minority stressors among LGBTQ+ young adults, findings from our study may inform research, practice and policies that could prevent and eliminate mental health inequities among LGBTQ+ young adults. Prevention interventions tailored for LGBTQ+ young adults suffering from psychological distress must address distinct classes and gradients of LGBTQ+-related minority stress.

## Supporting information

Shrader et al. supplementary material Shrader et al. supplementary material

## Data Availability

Data are available from the first author upon reasonable request to replicate analytic findings as reported in this paper.

## Supplementary material

The supplementary material for this article can be found at https://doi.org/10.1017/S2045796024000118.
