# Supplementary material for: Mental health impact of multiple sexually minoritized and gender expansive stressors among LGBTQ+ young adults: a latent class analysis
Source: Epidemiol Psychiatr Sci. 2024 Apr 11;33:e22. doi: 10.1017/S2045796024000118 (PMC11022265; doi:10.1017/S2045796024000118)
Supplement: Shrader et al. supplementary material [file S2045796024000118sup001.docx]

*Appendix 1: LGBTQ+-related Minority Stress Questionnaires*

*Familial Heterosexist Experiences Scale as adapted from 7-items from the Daily Heterosexist Experiences Questionnaire (DHEQ) (Balsam et al., 2013)*

**Please read each statement and let us know if it has happened to you in the past year (yes/no):**

1. Being rejected by my mother for being LGBTQ.
2. Being rejected by my father for being LGBTQ.
3. Being rejected by my legal guardian for being LGBTQ.
4. Being rejected by a sibling or siblings because I am LGBTQ.
5. Being rejected by other relatives because I am LGBTQ.
6. Family members not accepting your partner as part of the family.
7. Family members avoiding talking about your LGBTQ identity.

*LGBTQ+-Related Family Rejection Scale as adapted from 10-items from the “family rejection” subscale of the Sexual Minority Adolescent Sexual Minority Stress Inventory (SMASI) (Schrager et al., 2018)*

**Please read each statement and let us know if it has happened to you in the past year (yes/no):**

1. Hearing family members make negative comments about LGBTQ people.
2. Lying to my family about being LGBTQ.
3. If I come out, it will cause problems in my family.
4. My family does not want to talk to me about being LGBTQ.
5. Someone who lives with me has told me they disapprove of me being LGBTQ.
6. I feel as though I am a disappointment to my family because I am LGBTQ.
7. My family has told me that being LGBTQ is just a phase.
8. My parents are uncomfortable with LGBTQ people.
9. My parents are sad that I am LGBTQ.
10. My family tries to make me straight.

*Internalized LGBTQ+-Phobia Scale as adapted from 7-items from the LGBT Minority Stress Measure (LMSM) (Outland, 2016)*

**Please read each statement and let us know if it has happened to you in the past year (yes/no):**

1. If I was offered the chance to be someone who is not LGBTQ, I would accept the opportunity.
2. I wish I wasn’t LGBTQ.
3. I envy people who are not LGBTQ.
4. I feel that being LGBTQ is a personal flaw in me.
5. I feel that me being LGBTQ must have been a mistake of fate or nature.
6. I wonder why I am not “normal” and like everyone else.
7. I have tried to stop being LGBTQ.

*LGBTQ+ Identity Concealment Scale as adapted from 3-items from the LGBT Minority Stress Measure (LMSM) and 4-items from the Daily Heterosexist Experiences Questionnaire (DHEQ) (Outland, 2016; Balsam et al., 2013)*

**Please read each statement and let us know if you have done this in the past year (yes/no):**

1. Avoided telling people about certain things in my life that might imply I am LGBTQ.
2. Avoided talking about my romantic life because I do not want others to know I am LGBTQ.
3. Did not object when I heard anti-LGBTQ remarks because I did not want others to assume I am LGBTQ.
4. Watched what I said and did around heterosexual people.
5. Pretended that I had an opposite-sex partner.
6. Pretended that I was heterosexual.
7. Hid part of my life from other people.

*Psychological Distress Scale as measured by the 10-item Kessler-10 (K10) (Kessler et al., 2002)*

**Please read each statement and answer how often you have felt this way during the last 30 days (None of the time, A little of the time, Some of the time, Most of the time, All of the time):**

1. tired out for no good reason?
2. nervous?
3. so nervous that nothing could calm you down?
4. hopeless?
5. restless or fidgety?
6. so restless you could not sit still?
7. depressed?
8. so depressed that nothing could cheer you up?
9. that everything was an effort?
10. worthless?
